# Reflectance According to Cell Size, Foaming Ratio and Refractive Index of Microcellular Foamed Amorphous Polymer

**DOI:** 10.3390/ijms20236068

**Published:** 2019-12-02

**Authors:** Sung Woon Cha, Soo-hyun Cho, Joo Seong Sohn, Youngjae Ryu, Jeonghun Ahn

**Affiliations:** 1 School of Mechanical Engineering, Yonsei University, 50, Yonsei-ro, Seodaemun-gu, Seoul 03722, Korea; s00hyun@yonsei.ac.kr (S.-h.C.); ssamjjang87@yonsei.ac.kr (J.S.S.); yjryu1027@yonsei.ac.kr (Y.R.); 2 Molding Machines Division, LS Mtron, Anyang-si, Gyeonggi-do 14118, Korea; jeonghun.ahn@lsmtron.com

**Keywords:** microcellular foaming process, batch process, amorphous polymer, optical properties, foaming ratio, refractive index, diffused reflectivity

## Abstract

Microcellular foamed plastic has a cell size of approximately 0.1 to 10 microns inside a foamed polymer and a cell density in the range of 10^9^ to 10^15^ cells/cm^3^. Typically, the formation of numerous uniform cells inside a polymer can be effectively used for various purposes, such as lightweight materials, insulation and sound absorbing materials. However, it has recently been reported that these dense cell structures, which are induced through microcellular foaming, can affect the light passing through the medium, which affects the haze and permeability and causes the diffused reflection of light to achieve high diffuse reflectivity. In this study, the effects of cell size, foaming ratio and refractive index on the optical performance were investigated by applying the microcellular foaming process to three types of amorphous polymer materials. Thus, this study experimentally confirmed that the advantages of porous materials can be implemented as optical properties by providing a high specific surface area as a small and uniform cell formed by inducing a high foaming ratio through a microcellular foaming process.

## 1. Introduction

In modern industrial society, plastics are the most widely used materials owing to their various advantages, including good formability, low cost and high productivity. Although plastic was previously only recognized as an inexpensive, lightweight and rust-free material, recent technological innovations have shown that it is a high value-added material combining high performance and efficiency and its use has expanded into various industries. Accordingly, research into the process of developing and manufacturing new plastics for existing industrial uses has been actively conducted [1,2,3]. However, because plastics are based on fossil fuels, they are directly and indirectly related to fossil fuel depletion and environmental issues. Therefore, it is important to reduce the negative impact of materials and manufacturing processes.

One of the processes for manufacturing plastic molded products—namely, the foaming method—is a technology for forming micron cells inside the product. The microcellular foamed plastic is produced by the microcellular foaming process (MCPs), which was developed at the Massachusetts Institute of Technology (MIT) in the 1980s; the above-mentioned plastic is comprised of a cell size of 0.1 to 10 microns and a cell density in the range of 10^9^ cells/cm^3^ to 10^15^ cells/cm^3^ [4]. MCPs can reduce the amount of raw materials used in the same volume to achieve additional energy savings through the weight reduction effect. Further, it has been established that traditional foaming agents such as chloroflurocarbons (CFCs) can adversely affect the environment [5,6]. However, in the MCPs [7,8,9,10], inert gases, such as carbon dioxide and nitrogen are used as blowing agents to reduce the adverse environmental effects that may occur during the manufacturing process, form even finer cells and enable focus on environmentally friendly factors.

As research into the process of microcellular foaming continues, new facts about the structural features of micron cells are being discovered [11,12,13]. Hence, it is possible to further develop functional characteristics such as acoustic characteristics (sound absorption and sound insulation performance) and electrical insulation characteristics (change of dielectric constant) [14,15].

Additionally, the optical characteristics of microcellular foamed plastics have been applied to lighting technology such as Liquid Crystal Display (LCD) monitors [16]. They have been applied to television by forming a Back Light Unit (BLU) in the form of an optical sheet comprising a reflective sheet and diffusion sheet, wherein microcellular foaming technology is used to derive the reflection and diffusion performance required for such an optical sheet. According to previous research, the dense structure of the cells inside the microcellular foamed plastic affects the light passing through the medium, which in turn affects the light’s haze, permeability and diffuse reflection, which results in high diffuse reflectance [17,18,19,20,21]. This diffuse reflectance is higher than that of a conventional reflector using reflector filler insertion and surface patterning such as titanium dioxide (TiO_2_) and calcium carbonate (CaCO_3_).

Although the diffuse reflectances of a few microcellular materials have been studied previously, such as polycarbonate (PC) and poly-lactic acid (PLA), there are still several materials that need to be studied [18,19]. Therefore, in this study, we investigated the diffuse reflectances of three microcellular foamed materials; these included PC, amorphous polyethylene terephthalate (APET) and poly methyl methacrylate (PMMA), the latter two of which have not been studied before. The three amorphous materials were fabricated into sheet-shaped specimens and subsequently foamed by employing a microcellular batch process. Further, the foaming properties, cell morphology and diffuse reflectance were confirmed according to the respective conditions.

## 2. Results and Discussion

In this study, the variations in foaming and reflectance were investigated by controlling the foaming temperature of the three amorphous polymers, namely, APET, PC and PMMA. This is achieved by dissolving the gas inside the amorphous polymer through the microcellular foaming batch process and subsequent foaming. By applying these series of processes, cells with micron units were generated inside the foamed polymer material; this was aimed at altering the optical path and increasing the amount of scattered light. In this study, the gas was dissolved in the three amorphous polymers (APET, PC, PMMA) under the pressure of 5.5 MPa induced by carbon dioxide. In the foaming process after gas dissolution, glycerin was used to apply uniform heat to the specimen; further, the change in the foaming ratio was confirmed by altering the foaming temperature from 50 to 140 °C. The microcellular foamed plastics thus prepared were examined for their cell shape and distribution of the cross section through scanning electron microscopy (SEM); meanwhile, the diffuse reflection performance was measured by using a spectrophotometer.

### 2.1. Solubility

Solubility can be defined as the maximum concentration at which the polymer can dissolve the blowing agent. Moreover, solubility can vary depending on the conditions of the saturation process, such as the blowing agent type, phase, saturation pressure, saturation time and saturation temperature, which are generally non-linear [4,22,23]. Moreover, the solubility is positively correlated with the foaming ratio of the final product or test specimen. Therefore, to obtain a high foaming ratio, it is important to continuously maintain the solubility in the polymer. Consequently, the effect of the material properties on the solubility is being actively investigated.

Solubility [w.t.%] = (W_S_ − W_0_)/W_0._ (1)

The solubility can be calculated using Equation 1, where W_S_ is the mass of the polymer material specimen containing the blowing agent and W_0_ is the mass of the specimen before the saturation process. The solubility obtained by testing under the batch process conditions considered in this study is presented in Table 1.

### 2.2. Foaming Ratio

The foaming ratio is the most important factor in the MCPs and can be calculated based on the density change, as expressed by Equation (2) where D_0_ is the density of the polymer before foaming and D_f_ is the density of the microcellular foamed plastic produced after foaming. Therefore, this foaming ratio can be considered to represent the number of cells inside the microcellular foamed plastic.

Foaming Ratio [%] = (D_0_ − D_f_)/D_0._ (2)

**Figure 1:**
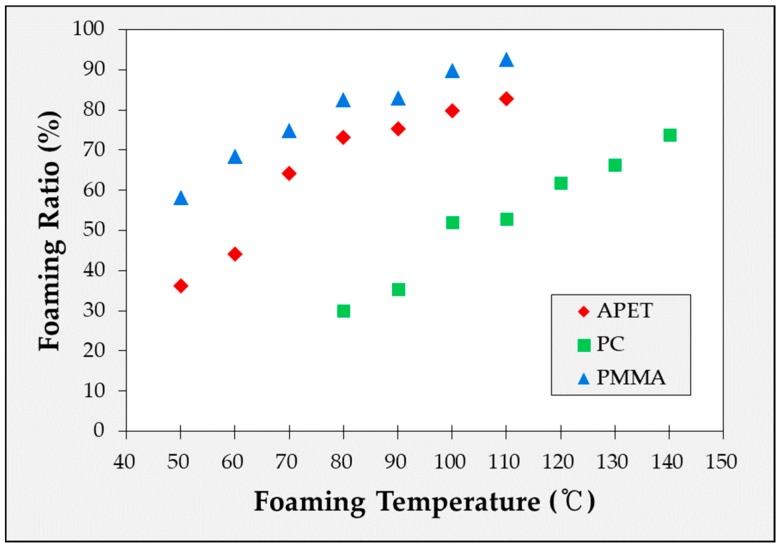
Foaming ratio of amorphous polyethylene terephthalate (APET), polycarbonate (PC) and poly methyl methacrylate (PMMA) according to foaming temperature.

The experimentally obtained foaming ratio is shown in Figure 1. Five or more experiments were conducted under each condition to obtain the specimens. In Figure 1, it can be seen that the foaming ratio increased with the foaming temperature, while the slope gradually decreased within the valid temperature range.

### 2.3. Cell Size Change with Foaming Temperature of Specimens with Equal Dissolution

**Figure 2:**
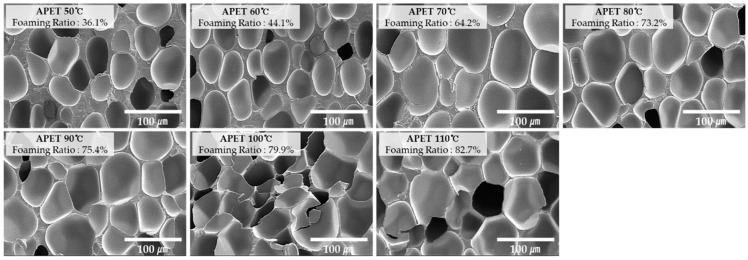
Cross-sectional scanning electron microscope (SEM) images (×400) of APET according to foaming temperature.

**Figure 3:**
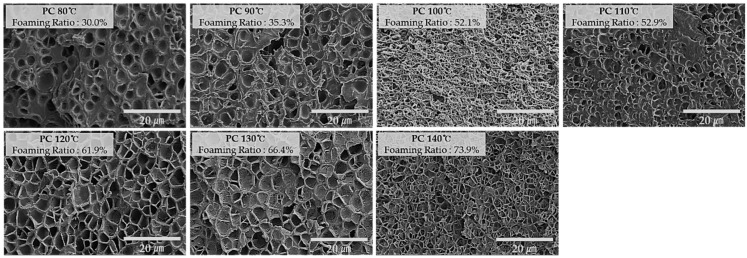
Cross-sectional SEM images (×2000) of PC according to foaming temperature.

**Figure 4:**
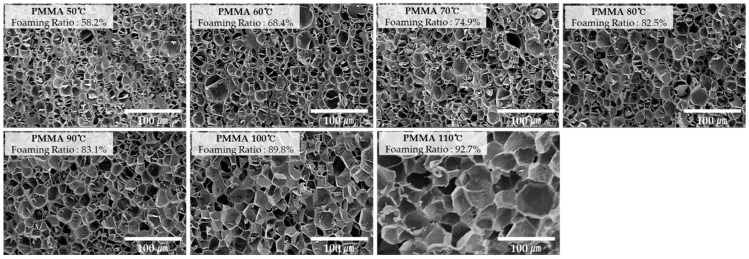
Cross-sectional SEM images (×400) of PMMA according to foaming temperature.

Figure 2, Figure 3 and Figure 4 show the scanning electron microscopy (SEM) of the specimen cross-sections prepared using the MCPs. In order to observe the cross section of the microcellular foamed plastic, the specimen was notched and subsequently cut by applying impact; meanwhile, the specimen was rapidly cooled down using liquid nitrogen. This was to ensure that no impairment is induced because cutting the specimen with scissors or knife can damage the structure of the cell as the cross section is pressed. Subsequently, the cross section of the specimen was coated with platinum (Pt) using a sputtering equipment. This suppresses the charging by forming a conductive metal layer on the cross section of the specimen; further, this coating reduces the thermal damage due to the energy of the electron beam and improves the secondary electron signal.

By examining the image, it can be seen that the cells have different shapes according to each material and the cell size and distribution are also different. 

### 2.4. Diffuse Reflectivity

#### 2.4.1. Diffuse Reflectivity According to Foaming Temperature of Microcellular Foamed Amorphous Polymer

**Figure 5:**
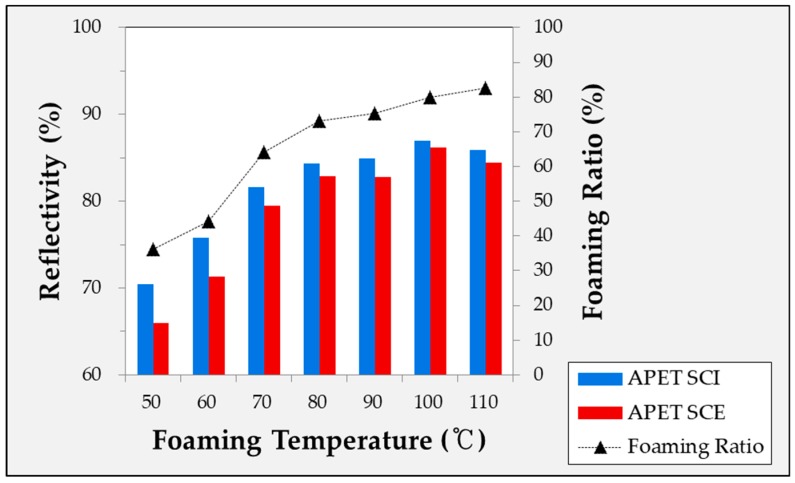
Specular reflectivity and diffuse reflectivity of microcellular foamed APET according to foaming temperature.

**Figure 6:**
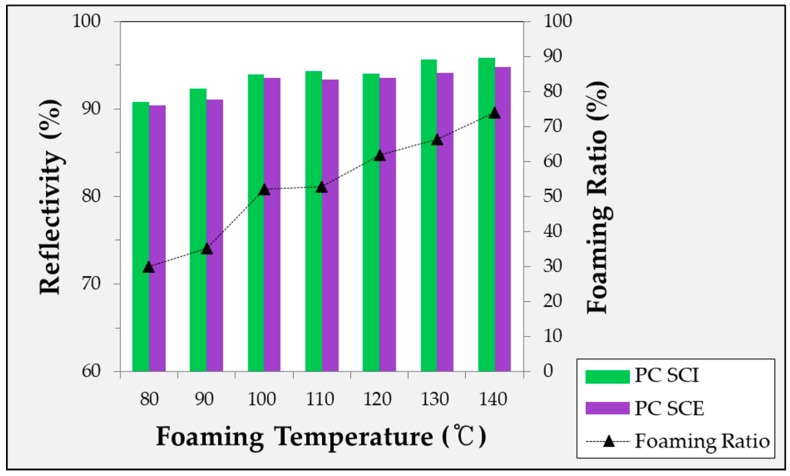
Specular reflectivity and diffuse reflectivity of microcellular foamed PC according to foaming temperature.

**Figure 7:**
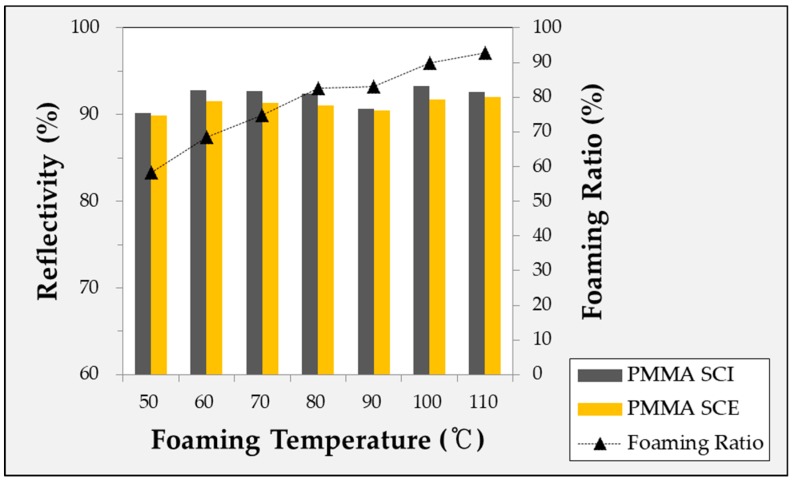
Specular reflectivity and diffuse reflectivity of microcellular foamed PMMA according to foaming temperature.

In Figure 5, Figure 6 and Figure 7, the reflectivity values of the microcellular foamed plastics according to the foaming temperature are divided into the specular component included (SCI) and specular component excluded (SCE) groups. The light that is incident on the surface of the object and reflected at the same angle is called specular reflectance, while the light scattered and reflected in various directions without being specular is called diffuse reflectance. The combination of specular and diffuse reflectance is called the total reflectance. Therefore, the SCI value is the total reflectance and includes the specular reflection, while SCE refers to the diffuse reflectance excluding the specular reflection in the total reflection. Therefore, under all conditions, the SCI value is greater than or equal to the SCE value.

#### 2.4.2. Diffuse Reflectivity According to Wavelength of Microcellular Foamed Amorphous Polymer

**Figure 8:**
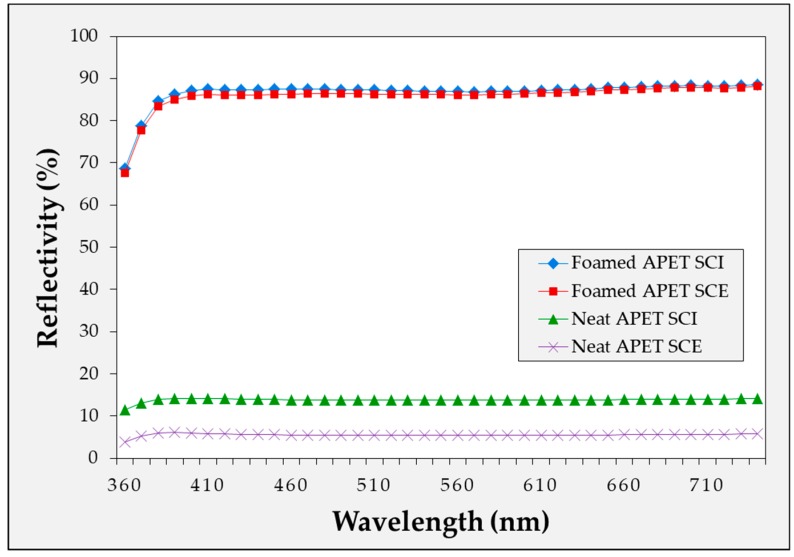
Diffuse reflectivity of microcellular foamed APET according to wavelength.

**Figure 9:**
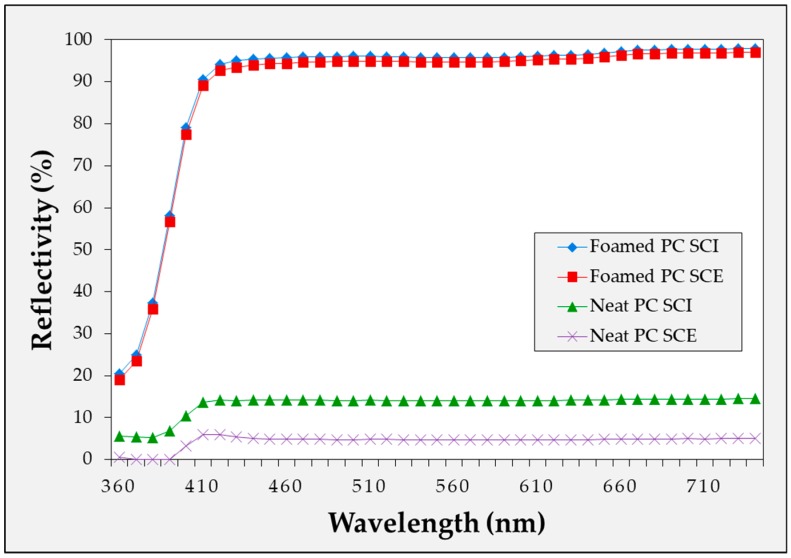
Diffuse reflectivity of microcellular foamed PC according to wavelength.

**Figure 10:**
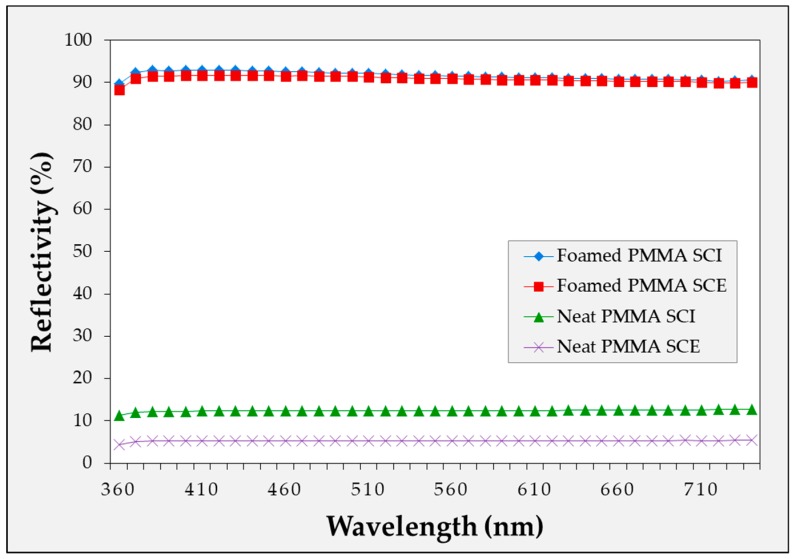
Diffuse reflectivity of microcellular foamed PMMA according to wavelength.

The diffuse reflectivity of the specimens with and without the MCPs was measured for each material as shown in Figure 8, Figure 9 and Figure 10. For the microcellular foamed specimens, the sample with the highest diffuse reflectance was selected under the experimental conditions and compared with the conditions prior to foaming. Thus, it was confirmed that the application of the MCPs resulted in a high level of reflectivity increase compared with the specimen before foaming. In particular, the amount of specular reflection was relatively high in the specimen before foaming, which indicates a large difference between the SCI and SCE values.

In this experiment, it was possible to confirm the difference in the reflectivity of each light wavelength according to the material. APET and PC had low reflectance in the low wavelength range of the visible light, while PMMA exhibited approximately uniform reflectance in the visible wavelength range. In the experiment, the difference in the diffuse reflectivity of each light wavelength according to the material was the light wavelength reflected by each material. Additionally, because the reflectivity tendency of each wavelength from the sample prior to foaming coincided with the reflectivity tendency of the wavelength after foaming, it was considered that the reflection characteristics were different for each material.

Blue light generated from devices, such as LED lighting, televisions, computers and smart phones, may be harmful to humans. Therefore, the problem may be solved by applying a diffuse reflection sheet made of a material such as PC with low reflectance in the low wavelength region to reduce the light with low wavelengths (mainly blue light).

## 3. Materials and Methods

### 3.1. Materials

In this study, three types of amorphous plastics, namely, APET (Taekwang Newtec, Inc. Seoul, Korea, Product Name: PLASTAR sheet by extrusion), PC (Taekwang Newtec, Inc. Seoul, Korea, Product Name: PLAGLAS sheet by extrusion) and PMMA (SPOLYTECH, Inc. Jincheon, Korea, Product Name: EXEET GLAS AG00 sheet by extrusion), were considered. Basic information on the above-mentioned materials is given in Table 2. Baldwin has reported that crystalline plastics and amorphous plastics have different nucleation characteristics [10]. Moreover, it is known that the temperature at which foaming occurs in amorphous plastics can be specified as the interval between the glass transition temperature and the melting temperature. Additionally, because amorphous plastics have fewer crystal regions than crystalline plastics, they can induce uniform foam throughout the entire region of the specimen, which is related to high and uniform diffuse reflectance. Hence, the three abovementioned materials were selected. The density and optical properties of the experimental materials are listed in Table 3.

### 3.2. Methods

#### 3.2.1. Microcellular Foaming Process

**Figure 11:**
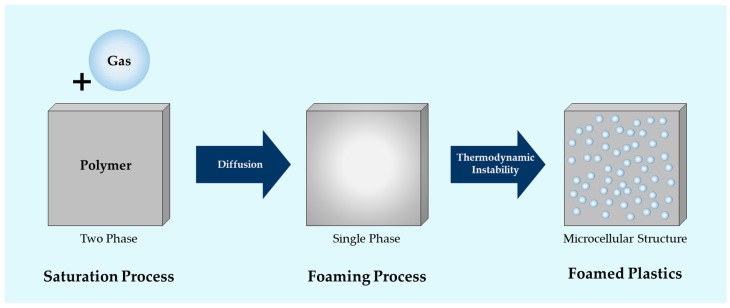
Mechanism of microcellular foaming process.

Microcellular foaming technology is used to form bubbles—called “cells”—within polymer materials. This technology uses the thermodynamic instability produced when a gas incorporated into a polymer material undergoes a rapid decrease in solubility. This MCPs mainly comprises two processes—namely, saturation and foaming—as shown in Figure 11. The saturation process dissolves inert gases, such as carbon dioxide or nitrogen, into the inner part of the polymer. To incorporate the gas into the inner part of the polymer, the sample is inserted into a high-pressure vessel and a high-pressure inert gas is injected at constant pressure for a certain amount of time. After saturation, when the sample with gas in its inner part is removed from the high-pressure vessel, the pressure decreases to that of ambient air. Then, the sample is heated, which rapidly reduces the solubility of the gas and creates thermodynamic instability that forms cells within the samples [24,25]. This process is known as foaming.

#### 3.2.2. Microcellular Batch Process

**Figure 12:**
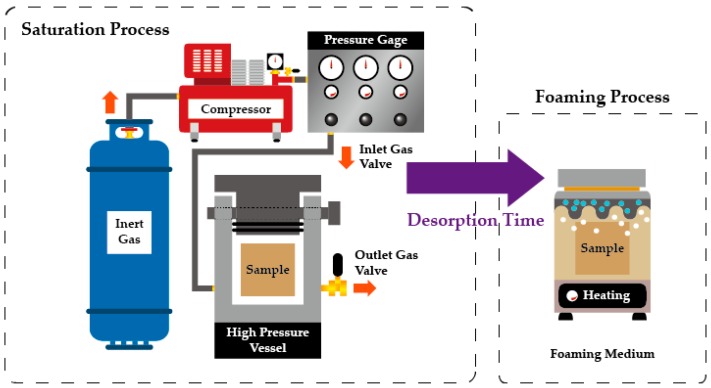
Schematic of microcellular batch process.

By using the batch process with a high pressure vessel, it is easier to control the process variables of the MCPs and obtain repeated test results, compared with a continuous process such as injection molding and extrusion molding. Additionally, it is easy to obtain the range of a low foaming ratio to a high foaming ratio and produce the corresponding specimen. Therefore, in this study, we selected the batch process to utilize these advantages and induce microcellular foaming. Figure 12 shows a schematic of the microcellular foaming batch process. The blowing agent was fed to a high pressure vessel through a compressor under certain pressure conditions. As mentioned above, the microcellular foaming batch process consisted of a saturation process, wherein the gas dissolved in plastic and a foaming process, wherein the dissolved gas grew into cells.

Firstly, in the saturation process, the temperature, pressure and time are controlled to saturate the blowing agent inside the plastic. Secondly, the foaming process is a process wherein foaming is carried out by applying a rapid pressure drop and high heat to change the solubility value, such that dissolved gas can grow into a cell and thereby cause thermodynamic instability. At this time, the type, temperature and time of the heat source applied to the specimen are controlled. Additionally, the time from the saturation process to the foaming process is defined as the desorption time. During this time, the specimen is kept under normal temperature and pressure and the dissolved gas inside the specimen is released into the air. Because this process can cause errors, the desorption time in the experiment must be controlled.

#### 3.2.3. Mechanism of Diffuse Reflection by Micron Cells

**Figure 13:**
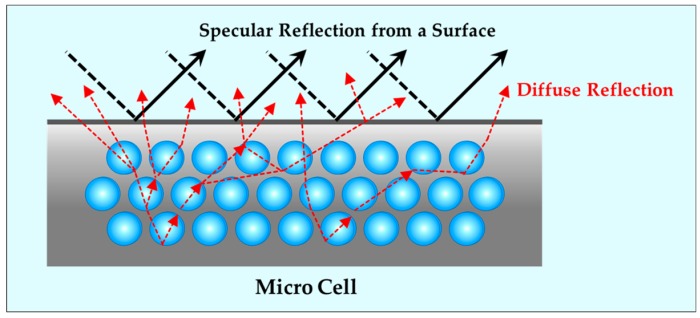
Mechanism of diffused reflection by micro cells.

Light reflected from the surface of an object appears in two forms, namely, diffuse and specular reflection. Specular reflection has a very high value at a certain angle, depending on the incidence angle of the light reflected on the surface, which is a reflection similar to the gloss of a metallic surfaces or mirrors. Diffused reflection refers to the degree to which the surface receiving the light reflects it in all directions. Diffuse reflection is independent of the incident light angle and the maximum value appears in the plane with normal perpendicular to the direction of the light source. As the specular reflection increases, the concentration of the reflected light also increases. A higher diffraction reflection component indicates that the reflected light forms over the entire reflecting surface, which means that the light is evenly distributed. The typical reflection plate performance can be measured by comparing the diffuse reflections. Figure 13 shows the reflection mechanism of the microcellular foamed plastic. When the light projected on the microcellular foamed plastic passes through the cell, a change of medium with different refractive indices occurs. Consequently, light reflection, transmission and absorption occurred. Particularly, the diffuse reflection of light occurs on the surface of the cell. This part of the projected light is reflected and the remaining part passes through the boundary between the cell and the plastic. The transmitted light repeats the abovementioned process in the cells of the other layer. Thus, the microcellular foamed plastic cell acts as a reflector to increase the diffuse reflectance of the material. Additionally, as discussed for the previous mechanism, the cells forming inside the microcellular foamed plastics exhibit a positive correlation between the foaming rate and the reflectance in the same material because the foamed structure itself acts as a reflector. However, previous studies [11,12,13,14] have demonstrated that, despite having the same foaming ratio, different cell shapes and distributions have different reflectances [17,18,19,20]. In other words, because the foaming rate is not the only factor affecting the reflectance, it should be considered in addition to other factors. In this study, samples with different cell shapes were fabricated to investigate the effects of cell shape, such as cell size and density effects and the effect of the micro foamed plastic’s refractive index on the reflectance. The change in the material and the reflectance of each wavelength were measured and confirmed. 

#### 3.2.4. Experimental Conditions

Table 4 lists the saturation conditions and desorption time of the MCPs. Other conditions were also set and tested. The blowing agent used carbon dioxide, which is an inert gas and the plastic specimens had a thickness of approximately 1.0 mm. The saturation time was set to approximately 24 h and preliminary experiments confirmed that the blowing agent saturated evenly inside the specimen after approximately 24 h. The saturation pressure was 5.5 MPa and the temperature was fixed at 27 °C. The desorption time, which is the time from the high pressure vessel to foaming, was set to 3 min to minimize the error caused by residual gas. Table 5 shows the conditions of the foaming process and quenching process. In the microcellular foaming batch process, pressure drop and temperature heating are performed to induce thermodynamic instability. In this study, glycerin was used as the foaming medium in the heating bath. It was selected as the medium as it has a higher boiling point than water; this was a necessity because it employed the use of a liquid to apply uniform heat to the specimen and had to further raise the temperature above 100 °C. The main experimental variables of the foaming process are the foaming temperature and time. The temperature required for foaming depends on the material, because when the gas molecules inside the material are energized and heated, movement activity occurs; when the material is heated, the thermal stiffness decreases. Thus, better foaming is achieved when more heat is applied. However, if the foaming temperature is excessively high, the material melts and does not support the formation of pores; therefore, the foaming is not properly implemented. Hence, the foaming temperature is determined by the material’s glass transitional temperature (T_g_) or melt temperature (T_m_). T_g_ is a characteristic transition that is observed in all crystalline and amorphous polymers. In general, at values of temperature below the T_g_, the polymer is in a solid state or a fragile glass phase; however, at temperatures higher than the T_g_, it is liquid or rubbery. In particular, the amorphous polymer does not have a T_m_; consequently, above the T_g_, gradual softening occurs as a result of the mobility of the polymer chain segment. Preliminary experiments confirmed the optimum foaming temperature range under the abovementioned saturation conditions. The foaming time is the time required for the specimen to be heated; if the foaming time is not sufficient, heat cannot be properly transferred to the center of the specimen. The foaming time was set to 30 s and the finished sample was cooled with water to prevent further effects caused by the changes in the specimen’s interior after the cell generation.

#### 3.2.5. Diffused Reflectivity

**Figure 14:**
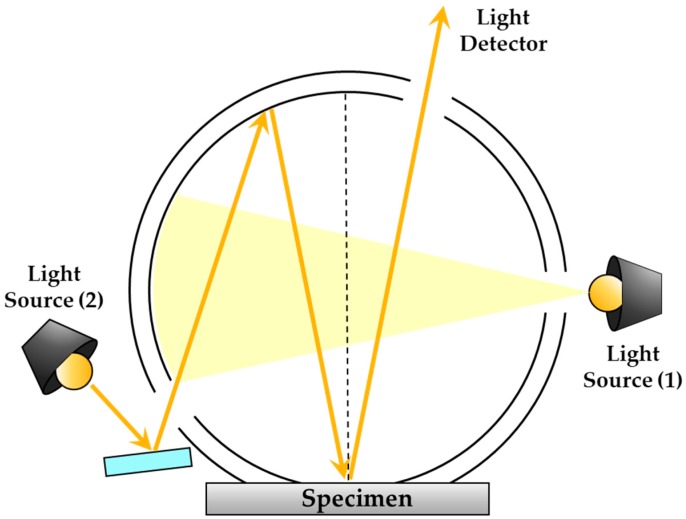
Schematic of device used to measure reflectivity; SCI/SCE simultaneous measurement method: Light sources (1) and (2) are located as illustrated at top. Light source (1) flashes first. This source is a normal diffused type and enables the user to obtain SCI measurement data when it is flashed. Subsequently, light source (2) flashes. This source enables numerical control of the speculary reflected light. The data obtained when this light source is flashed (i.e., the amount of light on the surface of the specimen) and that obtained when light sources (2) flashes can then be utilized to calculate the SCE measured data.

A spectrophotometer (KONICA MINOLTA Inc., Product No. CM-3600d, Tokyo, Japan) was used to measure the diffuse reflectance. This equipment can measure the transmittance, reflectance and color at 10 nm intervals in the visible wavelength range [26]. In this study, the reflection performance of the 550 nm wavelength, to which the human eye is considered to be most sensitive, was set as a representative value. When a light source is installed at a distance and certain angle to illuminate the specimen, the light reaches the plastic specimen and is reflected, permeated and absorbed. This equipment measures the intensity of the reflected light according to each wavelength to measure the reflectance. Figure 14 shows a schematic of the reflectance measuring device. A xenon lamp was used as the light source and the reflectance was measured in the visible wavelength range of 360–740 nm. Additionally, when measuring the diffuse reflectance, the total reflection component whose occurrence angle and reflection angle coincided, was excluded. The reflectance was expressed as a relative ratio after assuming that the reflectance of the barium sulfate (BaSO_4_) sample was 100%.

## 4. Conclusions

The results obtained in this study (Table 6) revealed that the foaming ratio tended to increase with the foaming temperature under the same saturation conditions. Additionally, the cell size tended to decrease depending on the foaming temperature but was not necessarily proportional. In general, when the cells are not adjacent to each other, the cell size increases as the foaming temperature increases. However, in the case of foamed specimens in which foam cells are densely foamed in close proximity with each other, the cells are bound to interfere with each other; further, it may also lead to an increase in the cell size due to expansion of the gas and enhance the fluidity depending on the temperature of the polymer material in which the gas is dissolved. Due to the above-mentioned reasons, it can be concluded that the cell size is not proportional to the foaming temperature. In order to reduce the size of the cell, there is a method of increasing the cell nucleation rate, which augments the saturation pressure of the gas. This result is consistent with the general trend of microcellular foamed polymers. Moreover, because the cell size was smaller, the reflectivity was expected to be higher because the refractive index difference between the matrix and the air (*n* = 1.00027784 of air at 550 nm) in the cell is induced several times. To summarize the results in Table 6, even if the cell size of the microcellular foamed polymer is small, if the foaming ratio is low, the distance between the cells causing the refractive index difference can be widened accordingly and the distance at which the incident light repeats the reflectance can be reduced. Therefore, the foaming ratio is an important factor at similar cell sizes. The PC with the highest reflectance exhibited the highest diffuse reflectivity because it had the smallest cell size and highest refractive index amongst the three materials used in the experiment. The microcellular foamed plastic exhibits excellent diffuse reflection performance; this is because the refractive index of the material is larger and the cell size is smaller. This is only possible when the foam is induced so densely that the cells are adjacent to each other. In a series of experiments with three different materials, it was difficult to determine the effect of the refractive index difference because the factors affecting the reflectance were coupled amongst them. Therefore, further studies and experiments should be performed in future work.

## Figures and Tables

**Table 1:** Solubility of amorphous polymer used in this study.

Materials	APET ^1^	PC ^2^	PMMA ^3^
Solubility (%)	6.4	11.4	21.5

^1^ APET (Amorphous polyethylene terephthalate), ^2^ PC (Polycarbonate), ^3^ PMMA (Poly methyl methacrylate).

**Table 2:** Specifications of the sheet used in this study.

Material Information	APET	PC	PMMA
Supplier	Taekwang Newtec	Taekwang Newtec	Spolytech
Grade Name	PLASTAR	PLAGLAS	EXEET GLAS AG00
Density (g/cm^3^)	1.29	1.19	1.18
Glass Transition Temperature (°C)	81	147	105
Melting Temperature (°C)	260	250	160

**Table 3:** Optical information of the amorphous polymer materials employed in this study.

Materials	Refractive Index	Reflectivity (%)	Transmittance (%)
APET	1.404	13.86	88.69
PC	1.586	13.73	90.13
PMMA	1.470	12.45	92.81

**Table 4:** Detailed experimental conditions for saturation and desorption time.

Experimental Conditions	Values
Blowing Agent	CO_2_
Material Thickness (mm)	1.0
Saturation Time (hours)	24
Saturation Temperature (°C)	27
Saturation Pressure (MPa)	5.5
Desorption Time (min)	3

**Table 5:** Detailed experimental conditions for foaming and quenching.

Experimental Conditions	Values
Foaming Medium	Glycerin (99% purity)
Foaming Temperature (°C)	50–140
Foaming Time (s)	30
Cooling Medium	Water
Cooling Temperature (°C)	10
Cooling Time (s)	60

**Table 6:** Foaming ratio, cell size and reflectance of microcellular foamed plastic according to material and foaming temperature.

**Material**	**Refractive Index**	**Foaming Temperature (°C)**		**50**	**60**	**70**	**80**	**90**	**100**	**110**
APET	1.404	Foaming Ratio (%)		36.1	44.1	64.2	73.2	75.4	79.9	82.7
		Cell Size (micron)		42.0	43.5	51.2	41.7	42.3	40.6	43.9
		Reflectivity (at 550 nm)	SCI (%)	70.4	75.8	81.6	84.3	84.9	87.0	82.7
			SCE (%)	66.0	71.3	79.5	82.9	82.8	86.2	84.
**Material**	**Refractive Index**	**Foaming Temperature (°C)**		**80**	**90**	**100**	**110**	**120**	**130**	**140**
PC	1.586	Foaming Ratio (%)		30.0	35.3	52.1	52.9	61.9	66.4	73.9
		Cell Size (micron)		3.0	3.8	1.6	2.4	2.7	3.2	1.8
		Reflectivity (at 550 nm)	SCI (%)	90.8	92.3	93.9	94.3	94.0	95.6	95.8
			SCE (%)	90.4	91.1	93.5	93.4	93.4	94.1	94.8
**Material**	**Refractive Index**	**Foaming Temperature (°C)**		**50**	**60**	**70**	**80**	**90**	**100**	**110**
PMMA	1.470	Foaming Ratio (%)		58.2	68.4	74.9	82.5	93.1	89.8	92.7
		Cell Size (micron)		18.4	21.2	22.0	23.5	21.8	24.3	31.8
		Reflectivity (at 550 nm)	SCI (%)	90.2	92.8	92.7	92.4	90.7	93.3	92.6
			SCE (%)	89.9	91.5	91.3	91.0	90.5	91.7	92.0

## Abbreviations

MCPs	Microcellular foaming process
APET	Amorphous polyethylene terephthalate
PC	Polycarbonate
PMMA	Poly methyl methacrylate
LCD	Liquid crystal display
BLU	Back light unit
SCI	Specular component included
SCE	Specular component excluded
